# Integrin α6Bβ4 inhibits colon cancer cell proliferation and c-Myc activity

**DOI:** 10.1186/1471-2407-9-223

**Published:** 2009-07-09

**Authors:** Anders Bondo Dydensborg, Inga C Teller, Jean-François Groulx, Nuria Basora, Fréderic Paré, Elizabeth Herring, Rémy Gauthier, Dominique Jean, Jean-François Beaulieu

**Affiliations:** 1 CIHR Team on the Digestive Epithelium, Department of Anatomy and Cell Biology, Faculty of Medicine and Health Sciences, Université de Sherbrooke, Sherbrooke, Québec, J1H 5N4, Canada

## Abstract

**Background:**

Integrins are known to be important contributors to cancer progression. We have previously shown that the integrin β4 subunit is up-regulated in primary colon cancer. Its partner, the integrin α6 subunit, exists as two different mRNA splice variants, α6A and α6B, that differ in their cytoplasmic domains but evidence for distinct biological functions of these α6 splice variants is still lacking.

**Methods:**

In this work, we first analyzed the expression of integrin α6A and α6B at the protein and transcript levels in normal human colonic cells as well as colorectal adenocarcinoma cells from both primary tumors and established cell lines. Then, using forced expression experiments, we investigated the effect of α6A and α6B on the regulation of cell proliferation in a colon cancer cell line.

**Results:**

Using variant-specific antibodies, we observed that α6A and α6B are differentially expressed both within the normal adult colonic epithelium and between normal and diseased colonic tissues. Proliferative cells located in the lower half of the glands were found to predominantly express α6A, while the differentiated and quiescent colonocytes in the upper half of the glands and surface epithelium expressed α6B. A relative decrease of α6B expression was also identified in primary colon tumors and adenocarcinoma cell lines suggesting that the α6A/α6B ratios may be linked to the proliferative status of colonic cells. Additional studies in colon cancer cells showed that experimentally restoring the α6A/α6B balance in favor of α6B caused a decrease in cellular S-phase entry and repressed the activity of c-Myc.

**Conclusion:**

The findings that the α6Bβ4 integrin is expressed in quiescent normal colonic cells and is significantly down-regulated in colon cancer cells relative to its α6Aβ4 counterpart are consistent with the anti-proliferative influence and inhibitory effect on c-Myc activity identified for this α6Bβ4 integrin. Taken together, these findings point out the importance of integrin variant expression in colon cancer cell biology.

## Background

Integrins are heterodimeric, transmembrane receptors composed of an α and a β subunit that transmit signals from extracellular matrix components to the cell interior. Integrins do not possess intrinsic signalling capacities, but rather mediate positional information by interacting with a large range of scaffolding proteins resulting in activation of several signalling molecules, such as Ras and PI3K, leading to subsequent activation of, among other molecules, JNK, Jun, Erk and CyclinD [1]. The net result of this integrin mediated intracellular signalling is control of cellular functions such as proliferation, migration, invasion and survival, all of which are pivotal events in cancer progression [2]. The existence of 18 α subunits and 8 β subunits leads to the formation of at least 24 distinct integrins, which are further diversified by extensive alternative splicing and post-translational modifications [3].

The accumulated findings of an association between high expression levels of the α6 integrin subunit and carcinoma cell invasion, metastatic capacity, apoptosis evasion and negative patient outcome [4-6] strongly argue in favor of a role for α6 containing integrins in human cancers. While the α6 subunit can dimerize with either β1 or β4 subunits, it preferentially dimerizes with the β4 subunit. In fact, in cells that express significant amounts of β4, such as human intestinal epithelial cells [7], the formation of α6β1 is nominal. Recent work from our laboratory has demonstrated an overall up-regulation of the expression of the β4 integrin subunit in primary tumors of the human colon [8] strongly supporting the notion that the α6β4 integrin is an important player in the migration and invasion of colon cancer cells [4,9]. These observations, taken together with the reported presence of this major laminin receptor at the invasive front of colorectal cancers [10], argue for an important role for the α6β4 integrin in colon cancer progression [11].

The α6 integrin subunit exists as two distinct variants, α6A and α6B, that differ in their cytoplasmic domains as the result of alternative splicing of a single exon [12]. Thus, the inclusion of an alternatively spliced exon results in the formation of the α6A variant, while exclusion leads to a reading frame shift, use of an alternative stop codon and formation of the α6B variant. These two variants show dissimilar spatial and temporal patterns of expression in various human organs and tissues [13,14]. For example, the α6A variant is exclusive to the mammary gland, basal keratinocytes and peripheral nerves, while the α6B variant is strongly favored in the kidney [13]. These distinct expression patterns, in combination with the conservation of the two variants in all mammalian species studied, suggest a biological importance for the existence of both molecules [3]. These arguments are supported by the divergent capacities of the α6A and -B subunits to initiate intracellular biochemical events, namely tyrosine phosphorylation of paxillin [15] and activation of the Ras-MEK-ERK pathway [16]. However, further evidence for the biological role of these α6 integrin splice variants is still lacking.

In the present study, we sought to establish the expression patterns and possible functions of the α6A and α6B integrin subunits in the normal human and adenocarcinoma cells of the colon. Our data show that these two variants are differentially expressed according to cell state. The A variant was predominant in the progenitor cells of the normal colon while the B variant was predominant in mature differentiated colonocytes. Significantly, we show a diminishment of α6B variant expression in primary colon carcinomas and adenocarcinoma cell lines. Interestingly, forced expression of the α6B variant revealed a specific ability of this variant to inhibit colonocyte proliferation and c-Myc activity.

## Methods

### Tissues

Samples of adult colon were obtained from patients between the ages of 49 and 86 years undergoing surgical treatment for colon adenocarcinoma. For each patient, samples from the primary tumor and from non-diseased areas (at least 10 cm distant from the lesion) corresponding to the resection margin were obtained. Diagnoses of adenocarcinoma were confirmed by pathologists. Staging of the carcinomas was according to Astler and Coller [17]. Resection margins of colon specimens obtained from patients undergoing surgery for pathologies other than colon cancer (bowel obstruction, diverticulosis, etc.) were also used for immunofluorescence. All tissues were obtained in accordance with protocols approved by the local Institutional Human Research Review Committee for the use of human material. The preparation and embedding of tissues for cryosectioning and RNA extraction was performed as described previously [18]. Additional paired samples (resection margin and confirmed carcinomas) were obtained from the Cooperative Human Tissue Network (Midwestern Division, Ohio State University, OH) which is funded by the National Cancer Institute.

### Indirect immunofluorescence

Cryosections 3 μm thick were fixed in 2% paraformaldehyde (α6A, Ki67 and Rbm19) or -20°C ethanol (α6B). Nonspecific protein-protein interactions were blocked for one hour at room temperature by immersion of slides in 10% goat serum (α6A) or 2% BSA (α6B) in PBS followed by incubation with the primary antibodies diluted 1:200 in their respective blocking solutions overnight at room temperature. Following extensive washing in PBS, the slides were incubated with either FITC or rhodamine conjugated secondary antibodies raised against mouse and rabbit IgG (Chemicon), respectively, for one hour at room temperature before being washed in PBS. The slides were stained with Evan's blue (0.01% in PBS) before being mounted in glycerol:PBS (9:1) containing 0.1% paraphenylenediamine and observed for fluorescence with a Leica Reichart Polyvar 2 microscope (Leica Canada, Saint-Laurent, QC) equipped with a Leica DFC300 FX digital color camera. In all cases, no immunofluorescent staining was observed when a mix of mouse and rabbit non-immune sera replaced primary antibodies.

### Primary antibodies

Two antibodies recognizing integrin α6A (1A10) and α6B (6B4) [13] were originally generous gifts from Dr. A. Sonnenberg (Division of Cell Biology, The Netherlands Cancer Institute, Amsterdam, The Netherlands). Subsequently, these antibodies were obtained from Chemicon (Temecula, CA; 1A10) and MUbio Products (Maastricht, The Netherlands; 6B4). Mabs 1A10 and 6B4 were used for western blots and co-immunoprecipitation. For indirect immunofluorescence, 6B4 and a rabbit polyclonal α6A (α6-cytoA) [19] antibody was obtained and employed in place of 1A10. This antibody was a generous gift from Dr. de Curtis (Department of Molecular Pathology and Medicine, San Raffaele Scientific Institute, Milan, Italy). The anti-Ki67 monoclonal antibody KiS5 and the polyclonal anti-lysozyme antiserum were from Chemicon and DAKO Cytomation (Glostrup, Denmark). The anti-progenitor cell Rbm19 antibody [20] was a kind gift from Dr. Alan M. Mayer (Department of Pediatrics, Medical College of Wisconsin, WI). To probe for β-actin, the antibody C4 from Chemicon was employed.

### Plasmids and plasmid construction

The c-Myc responsive luciferase reporter plasmid, pMyc-TA-Luc (Clontech, Mountain View, CA), carries six c-Myc binding sequences in front of the minimal TATA box from the herpes simplex thymidine kinase (HSV-TK) promoter. An Rb responsive luciferase reporter plasmid, pRb-TA-Luc (Clontech) was also used. An expression vector containing the cDNA of integrin α6A, pRc/CMV-α6A [21], was a generous gift from Dr. Sonnenberg (Division of Cell Biology, The Netherlands Cancer Institute, Amsterdam, The Netherlands). The α6A cDNA was subcloned into the viral expression vector pLPCX (BD Bioscience Clontech, Mississauga, ON) by a non-directional strategy using *Hind *III. Correct orientation was verified by restriction enzyme analysis. cDNA originating from a preparation of fetal epithelial enterocytes separated from the mesenchyme using Matrisperse (BD Biosciences, Mississauga, ON) as described previously [22] was used as a template for PCR amplification of the cytoplasmic tail of the α6B subunit using *Pwo *polymerase (Roche, Laval, QC). The upstream primer (5' TGCTGAAAGAAAATACCAGA 3') spanned an endogenous *Xba*I site, while the downstream primer (5' GC TCTAGAGAAAAAGCAGTTTGGGTACT 3') introduced another (underlined sequence.) The amplified DNA was ligated into pPCR-Script (Stratagene, La Jolla, CA) and verified for fidelity by sequencing. Subsequently, the cDNA encoding the cytoplasmic tail of the integrin α6A subunit in pRc/CMV-α6A was replaced by the cDNA encoding the cytoplasmic tail of the integrin α6B subunit by *Xba*I digestion of the recipient (pRc/CMV-α6A) and donor (pPCR-Script-α6B) vectors followed by ligation, generating pRc/CMV-α6B. The cDNA encoding the integrin α6B subunit was subcloned into the pLPCX vector using the same strategy as for α6A. A mammalian episomal expression vector, pEEP1, was used to generate populations of Caco-2/15 cells over-expressing the two integrin α6 splice variants. Integrin α6A or α6B subunit cDNA was excised from pRc/CMV-α6A and pRc/CMV-α6B, respectively, using *Hind*III, Klenow filled and blunt-end ligated into a Klenow filled *Not*I site in pEEP1, generating pEEP1-α6A and pEEP1-α6B.

### Cell culture and generation of colon cancer cells over-expressing α6A and α6B

The colon cancer cell line, Caco-2/15 was grown in DMEM (GIBCO, Burlington, ON) supplemented with 10% fetal bovine serum (ICN Biomedicals, Aurora, OH), 1% HEPES and 1% Glutamax (both from GIBCO, Burlington, ON) as described previously [23]. The colon cancer cell lines HT-29, COLO 201, DLD-1, HCT 116, T84, SW480 and SW620 were grown in accordance with instructions provided by the ATCC (Rockville, MD). All cells were grown in an atmosphere of 95% air and 5% CO_2 _at 37°C.

The pEEP1-α6A and pEEP1-α6B plasmids were introduced into 1 × 10^6 ^Caco-2/15 cells by nucleofection using the Amaxa Biosystem Nucleofection kit (ESBE Scientific, St. Laurent, QC) using the T-20 setting. Immediately following nucleofection, the cells were seeded onto collagen coated cell culture dishes (Falcon, Franklin Lakes, NJ). 24 hours post-nucleofection the cells were subjected to hygromycin (Multicell, St. Bruno, QC) selection at a concentration of 200 μg/ml for 10 days. This selection pressure was maintained throughout the experimental period to ensure continuous replication and transfer of the episomal plasmid. Forced expression of the α6A and α6B subunits was monitored by western blot.

### BrdU incorporation and staining

BrdU incorporation and staining was performed according to the manufacturer's (Roche Applied Science, Laval, QC) instructions. Briefly, 24 hours after plating of cells in LabTeks (Nalge Nunc, Rochester, NY) the cells were incubated for two hours with normal medium containing BrdU then immediately subjected to anti-BrdU and DAPI staining. For each condition, two random fields per well were counted for DAPI and BrdU positive cells and the BrdU positive cell population was determined as a percentage of total DAPI positive cells. All experiments were performed in quadruplicate and repeated three times.

### Western blot and immunoprecipitation

Western blots were performed as SDS-PAGE under non-denaturing conditions using 120 μg of whole cell lysate per lane. After transfer of the separated samples to a nitrocellulose membrane (BioRad, Hercules, CA) unspecific protein binding to the membrane was blocked by 5% skim milk-powder in PBS-0.1% Tween followed by incubation with the α6A 1A10 monoclonal antibody. Following detection, the membrane was stripped of antibody by incubation in stripping solution (50 mM Tris (pH 6.8), 2% SDS, 100 mM β-mercaptoethanol) at 50°C for 20 minutes after which the membrane was reprobed with the α6B 6B4 antibody using 2% BSA/0.1% Tween as blocking solution. Finally, the membrane was restripped and reprobed with a β-actin antibody as input control.

For immunoprecipitation of α6β4 and α6β1, newly confluent Caco-2/15 cells and keratinocytes (a kind gift of Dr L. Germain, LOEX, Université Laval, Québec, QC) were metabolically labeled using Promix [^35^S]methionine and cystine (Amersham Pharmacia Biotech), 200 μCi/ml for 6 h. Cells were lysed and processed as previously described [7] for immunoprecipitation of α6-containing integrins with the antibody G0H3 and Protein-G Sepharose (Invitrogen). Radioactive samples were analyzed under reduced and nonreduced conditions by SDS PAGE [7].

### RT-PCR

First strand cDNA synthesis was performed with 2 μg total RNA using oligo(dT)_12–18 _(Amersham Pharmacia, Bay d'Urfé, QC) as primer and Omniscript reverse transcriptase (Qiagen, Mississauga, ON) for synthesis. Primers used to co-amplify the α6A and α6B transcripts using 1/50 of the synthesized cDNA above were sense: 5'-CTAACGGAGTCTCACAACTC-3' and antisense: 5'-AGTTAAAACTGTAGGTTCG-3'. Each cycle was composed of template denaturation at 94°C for 1 minute, primer annealing at 65°C for 1 minute and elongation at 72°C for 1 minute. The primer annealing temperature was decreased by 0.5°C after each round of amplification for 40 cycles followed by a final 15 cycles at an annealing temperature of 45°C.

### Real-time quantitative RT-PCR

Quantitative RT-PCR was performed as previously described)[24]. Three different primer pairs for the integrin α6 subunit were tested for amplification efficiency and fidelity. The primer pair termed α6PD-2 was chosen for amplification of cDNA coding for the integrin α6 subunit based on a superior amplification efficiency and lack of primer dimer formation as assessed by melting curve analysis. The Ct-values were converted into relative expression values compared to a pooled RNA standard (QPCR Human Reference Total RNA, Stratagene, La Jolla, CA) before normalization of α6 expression against a weighted average of three normalizing genes (B2M, MTR & MAN1B1) using the geNorm applet [25]. Briefly, this algorithm normalizes a gene of interest against several normalizing genes, rather than against a single gene, thus obtaining an analysis of expression that is less likely to be impacted by any random fluctuations in the expression level of the normalizing gene(s). The sequences of the α6PD-2 primer were: sense 5' TGGGATATGCCTCCAGGTT 3', antisense 5' TGTAGCCACAGGGTTTCCTC 3'. Primer pairs for B2M, MAN1B1, and MTR have been described previously)[24]. The annealing temperatures of the reactions were 57°C (α6) or 58°C (B2M, MAN1B1 and MTR) and the amplification efficiencies of the reactions were 100.7%, 105.7%, 98.9% and 96.8% for α6, B2M, MTR and MAN1B1, respectively, as determined by standard curve analysis.

### Transfection and luciferase measurement

Equal numbers of Caco-2/15 cells were seeded in 24-well plates (Falcon, Franklin Lakes, NJ) and grown to 40–60% confluence before being transiently transfected in serum-free medium using FuGENE transfection agent (Roche, Indianapolis, IN) in a μg DNA to μl transfection agent ratio of 1:9. Cells were kept under normal growth conditions after transfection and harvested for analysis 48 hours post-transfection. All transfections were performed as co-transfections using a renilla luciferase expression plasmid to establish an internal control for transfection efficiency; thus allowing for normalization of cell number and viability. Promoter activities of the various reporter plasmids were expressed using the arbitrary unit "RLU" (relative luciferase units). Numeric values of CMV promoters in control transfections (empty vector) were kept equal to experimental (α6A and α6B) numeric values by adjusting the absolute level of plasmid as measured by μg. DNA concentrations in transfections were kept constant with the addition of pBluescript SK+ (Stratagene, Cedar Creek, TX). Equal amounts (25 ng) of reporter plasmid and expression vector (pRc/CMV-α6A and pRc/CMV-α6B) were cotransfected with 2 ng of pCMV-Renilla per well. Firefly and renilla luciferase activity was measured using the Dual-Luciferase^® ^Reporter Assay System (Promega Corporation, Madison, WI) according to the manufacturer's instructions using an Orion microplate luminometer from Berthold (Montreal Biotech, Kirkland, QC) for detection of the chemiluminescent signal. Individual experimental results were normalized to the average of the RLU of the empty vector cotransfectant in the corresponding experiment.

## Results

### Expression of α6 subunit variants in the normal colon

**Figure 1:**
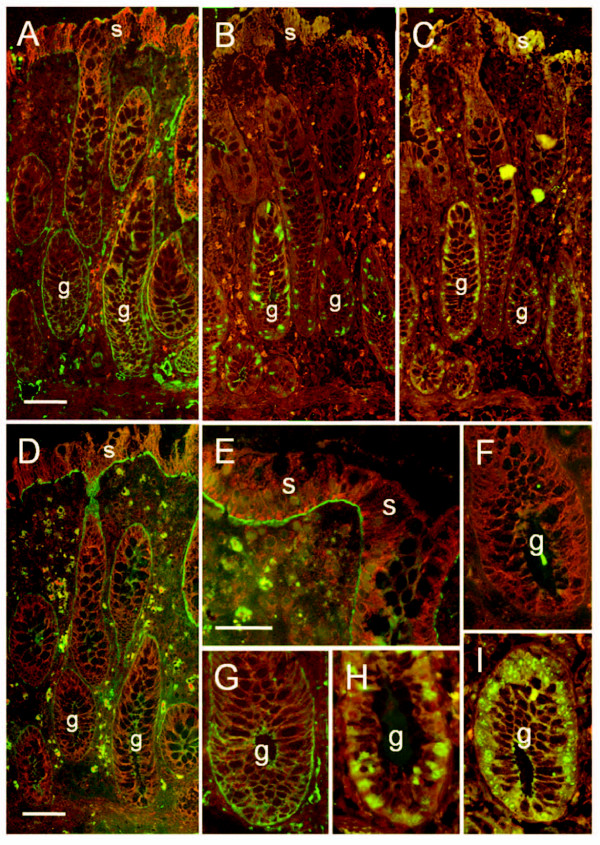
**Representative immunofluorescent staining of frozen serial sections of the adult colonic mucosa for the detection of the α6A and α6B splice variants and proliferation markers**. (A) Expression of the A variant showing a predominant distribution in the lower half of the glands (g), the region containing the progenitor cells as confirmed by immunodetection with Ki67 (B) and Rbm19 (C). (D, E) The B variant was detected in the upper half of the glands and at the surface (s) epithelium. (F-I) Higher magnification of the lower gland region stained for the detection of α6B (F), α6A (G), Ki67 (H) and Rbm19 (I) showed that in the colon, α6B is absent from the lower crypt while both α6A and the proliferative zone extend to the bottom of the glands. Red-brown signal: Evan's blue counter stain. Magnifications: A-D: scale bars in A and D = 50 μm; E-I: scale bar in E = 25 μm.

We previously showed the ubiquitous presence of the α6 dimerization partner β4 along the glandular unit of the human colon [8]. We, therefore, proceeded directly to delineate the specific expression patterns of the α6A and α6B splice variants in the normal human adult colonic mucosa (Figure 1). The α6A subunit was expressed in the basal membrane of epithelial cells concentrated in the lower half of the glands (Figure 1A), a region that contains the progenitor cells as identified by co-immunodetection with the proliferative antigens Ki67 and Rbm19 (Figure 1B, C, G, H, I). Staining for the α6B subunit on serial sections revealed its presence in the epithelium located in the upper half of the glands and at the base of the surface epithelium (Figure 1D, E), while it was not detected in the lower crypt (Figure 1F). The association of α6A with the actively growing cell population and α6B with the mature differentiated population supports the hypothesis that each variant possesses a specific distinct function, necessary for intestinal homeostasis.

### α6 is up-regulated in colon cancer cells and undergoes a shift away from the α6B variant

**Figure 2:**
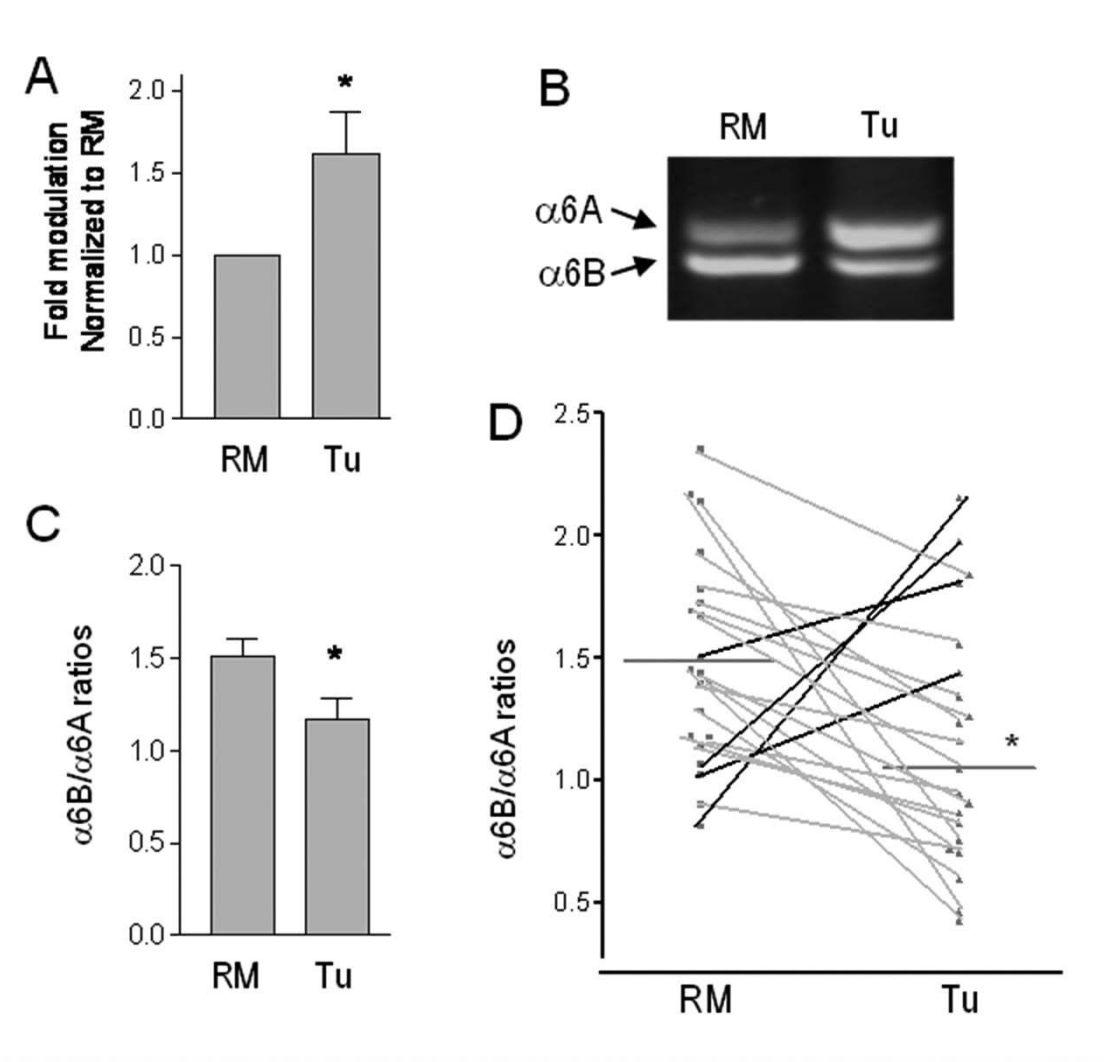
**Integrin α6 is up-regulated in colon cancer cells and undergoes a shift towards the α6A variant**. (A) Quantitative RT-PCR of total α6 subunit mRNA in patient matched resection margins (RM) and corresponding primary tumors (Tu). Mean ± SEM. *: *p *= 0.025, *n *= 21, paired t-test. Representative competitive RT-PCR of the α6A and α6B variants (B) and (C) ratio of α6B/α6A transcript levels in patient matched RM and primary tumors of the human colon. Mean ± SEM. *: *p *= 0.05, *n *= 21, paired t-test. (D) Dot graphs of the individual α6B/α6A ratios showing a sharp decrease in 17 (grey) of the 21 paired samples analyzed.

The α6β4 integrin is frequently up-regulated in several cancer types [2]. As in our previous study on the β4 subunit [8], using quantitative PCR we observed a significant up-regulation of total α6 expression in paired primary tumor samples versus patient matched normal resection margins (RM) (Figure 2A). We then assessed whether modulation of α6 splice-variant expression accompanied the overall up-regulation of total α6 in primary tumors. Competitive RT-PCR using primers that amplify the transcripts of both α6 variants showed an overall dominance of α6B expression in the healthy resection margins (Figure 2B – RM). Furthermore, a clear down-regulation of expression of the α6B variant in conjunction with a possible increase in the α6A variant were observed in patient matched tumors (Figure 2B, C). Overall, 81% of the 21 pairs showed the same change in expression in the tumor sample, resulting in a statistically significant shift towards a diminished α6B/A-ratio in primary cancers compared to healthy resection margins (Figure 2D). However, no correlation was noted between tumor grade or stage and α6B down-regulation (data not shown). A predominant expression of the α6B variant in normal colonic tissue is in accordance with the fact that the quiescent cell population outsizes the proliferative cell population in the normal colon. Correspondingly, a shift towards higher expression of the A variant in hyperproliferative colon carcinomas correlated with the association of this variant with the proliferative compartment of the healthy colon.

**Figure 3:**
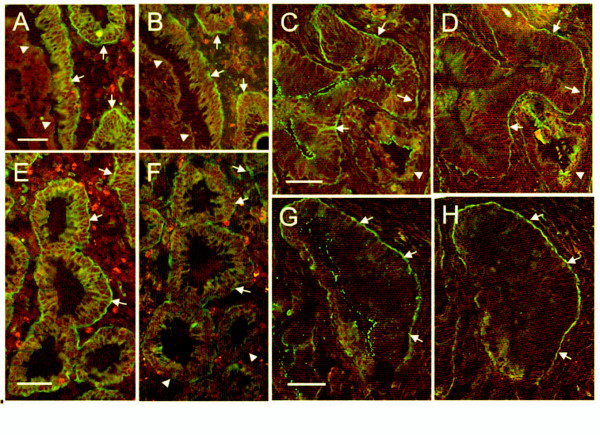
**Expression of α6A and α6B integrin subunits in colon cancer specimens**. Representative images by indirect immunofluorescence staining of α6A (A, C, E and G) and α6B (B, D, F and H) integrin subunits in serial sections of colon cancer specimens (A, B; C, D; E, F and G, H) showing the loss of variant segregation, leading to widespread overlap of both variants (arrows). Some regions, however, displayed the expression of only one variant, with no expression of the other (arrowheads). Red-brown signal: Evan's blue counter stain. Magnifications: scale bars = 50 μm.

The patterns of expression of α6A and α6B were analyzed *in situ *by indirect immunofluorescence on 8 primary tumors and their corresponding resection margins (Figure 3). All specimens were well- to moderately-differentiated tumors. In all examined resection margins the differential distribution of α6A in the lower half of the gland and α6B in the upper half of the gland and in the surface epithelium was comparable to that observed in normal colon specimens (data not shown). Analyses of frozen serial sections from primary tumors revealed that the A variant was now found almost ubiquitously expressed at the base of carcinoma cells (Figure 3A, C, E, and 3G) while the B variant was detected in most, but not all, corresponding regions (Figure 3B, D, F and 3H). These results exposed the widespread overlap of expression between the two α6 variants in colon cancer epithelia suggesting the loss of segregated expression found in normal colon tissue. In addition, extensive expression of α6A in carcinoma cells was consistent with the observed up-regulation of α6A at the transcript level (Figure 2B, D).

**Figure 4:**
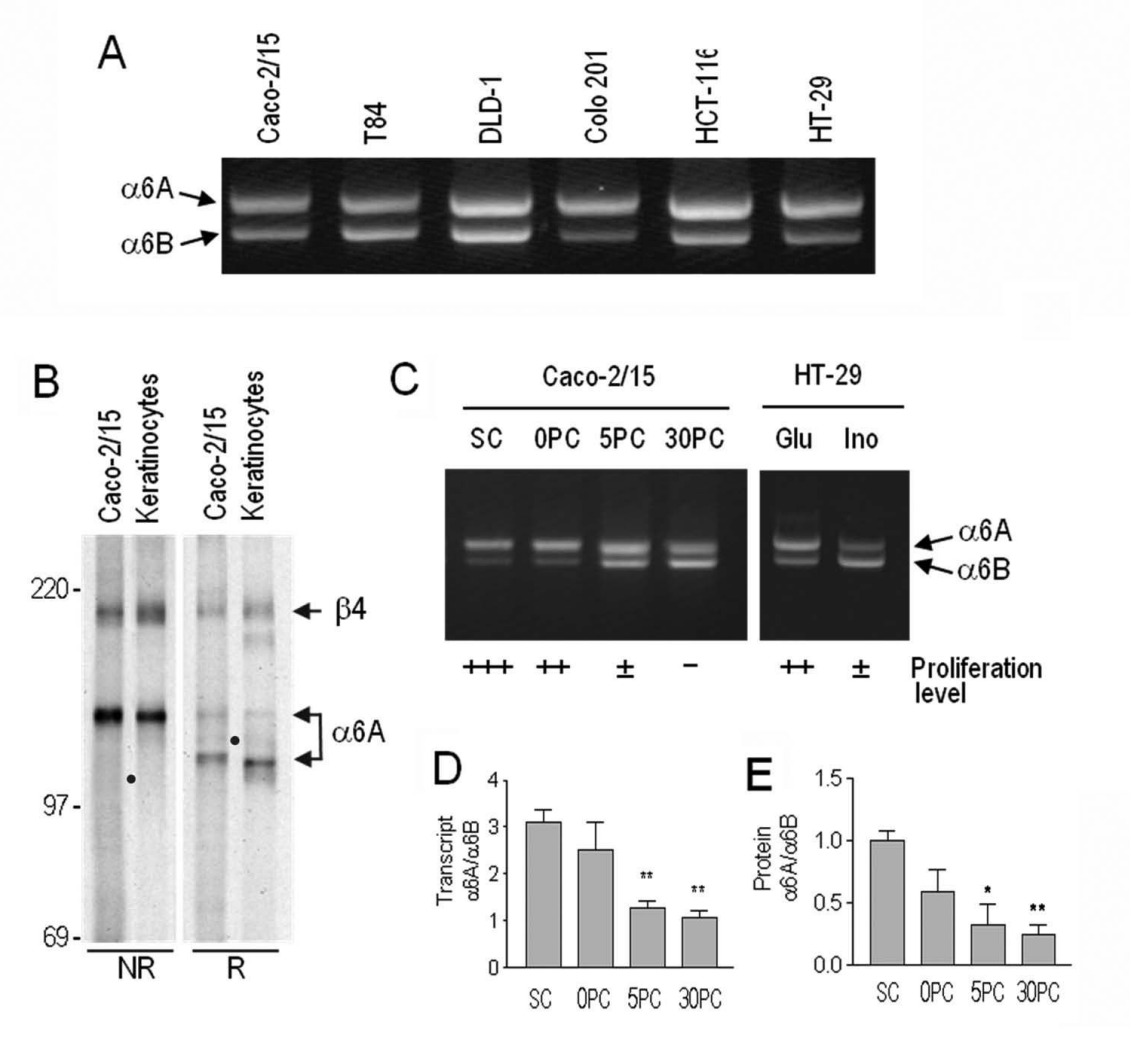
**Analysis of α6A and α6B integrin subunits in colon cancer cell models**. (A) Representative competitive RT-PCR of the α6A and α6B variants in six colon cancer cell lines. (B) Immunoprecipitation of the α6 subunits using the G0H3 antibody from metabolically labeled Caco-2/15 cells and keratinocytes and analyzed on SDS-PAGE under nonreduced (NR) and reduced (R) conditions showing that α6 predominantly associates with the β4 subunit in these cells. Apparent molecular weights are indicated on the left side. The dots indicate the expected sites of β1 subunit migration under both NR and R conditions. (C) Further analysis by competitive RT-PCR for splice variant expression of two intestinal cell lines at different differentiation stages showing up-regulation of the α6B variant upon cell-cycle exit. Caco-2/15: proliferative at sub-confluence (SC) while becoming non-proliferative at post-confluence (PC); HT-29: grown under non-permissive (Glu: glucose) or permissive (Ino: inosine) conditions for differentiation and cell cycle exit. (D, E) The α6A/α6B ratio of transcript (D) and protein (E) levels relative to differentiation of the various intestinal cells. Means ± SEM, n = 3–6. *, **: Statistically significantly different with *p *< 0.05 and 0.01, respectively, from subconfluent Caco-2/15. Tukey's One Way Analysis of Variance (ANOVA).

We next investigated the relative α6A and α6B levels in 6 well established colon cancer cell lines to see if this characteristic was conserved. All six cell lines tested predominantly expressed the A-variant (Figure 4A). However, at least one exception to this exists since the Colo 320 cell line has been previously shown to predominantly express the B-variant [13] suggesting that, as observed herein for the primary tumors where 4 out of the 21 pairs analyzed did not show an α6A subunit increase (Figure 2D), the relation between high α6A expression and a pro-proliferative state is not absolute in colon cancer cell lines of human origin although quite frequent. To further investigate the phenomenon, we used the two colon cancer cell lines, Caco-2/15 and HT-29 that share the trait of undergoing a differentiation program, characterized by a significant decline in proliferation, as a consequence of culture method. These two cell types express both β1 and β4 subunits but because the affinity of α6 for β4 is much greater than for β1, it can be assumed based on studies performed on keratinocytes and other cell types that the α6A/B variants mainly combine with β4 [7,13,26]. This was confirmed by immunoprecipitating the α6B complex from metabolically labeled Caco-2/15 or primary keratinocytes with [^35^S] methionine and cystine using the α6 subunit-specific G0H3 antibody and analysis by SDS-PAGE under reduced and non-reduced conditions. As shown in Figure 4B, both keratinocytes and Caco-2/15 cells display bands at approximately 205 kD and 150 kD and 205 kD and 120 kD corresponding to the β4 and α6 subunits under non-reduced and reduced conditions, respectively. The β1 subunit remained below detection level in Caco-2/15 cells confirming that α6 predominantly associates with β4 in intestinal cells. We thus used the two intestinal cell models Caco-2/15 and HT-29 to monitor the expression of the α6A and B variants during the course of proliferation arrest accompanied by the induction of differentiation. Competitive RT-PCR revealed that the ratio of α6A to α6B was altered as a function of cell state, reflecting the situation *in vivo*. Higher levels of α6A were associated with actively growing cells and a subsequent switch in favour of α6B was found to be associated with cells that had undergone cell cycle arrest (Figure 4C, D). This trend was mirrored in protein levels as well when detected with variant specific antibodies (Figure 3E).

### α6B inhibits proliferation in colon cancer cells

Elevated levels of unligated integrins can provoke intracellular signalling resulting in apoptosis [27]. Consequently, we focused our experiments on the well-characterized colon cancer cell line Caco-2/15 which has the ability to constitutively deposit significant amounts of laminins [28], the α6β4 ligands. To evaluate the hypothesis that an altered α6A/α6B ratio could be of functional importance for the proliferative status of colon cancer cells we attempted to create stable cell lines over-expressing α6A and α6B. However, we were repeatedly unable to maintain the α6B cells in long term cultures suggesting that over-expression of the α6B variant impaired cell proliferation. We were unable to perform complementary studies using RNAi since the only difference between the mRNA of the α6A and α6B variants is the inclusion of an alternatively spliced exon in the α6A transcript. As no region of the transcript is unique to the α6B variant it was not possible to exclusively target the α6B transcript for RNAi.

**Figure 5:**
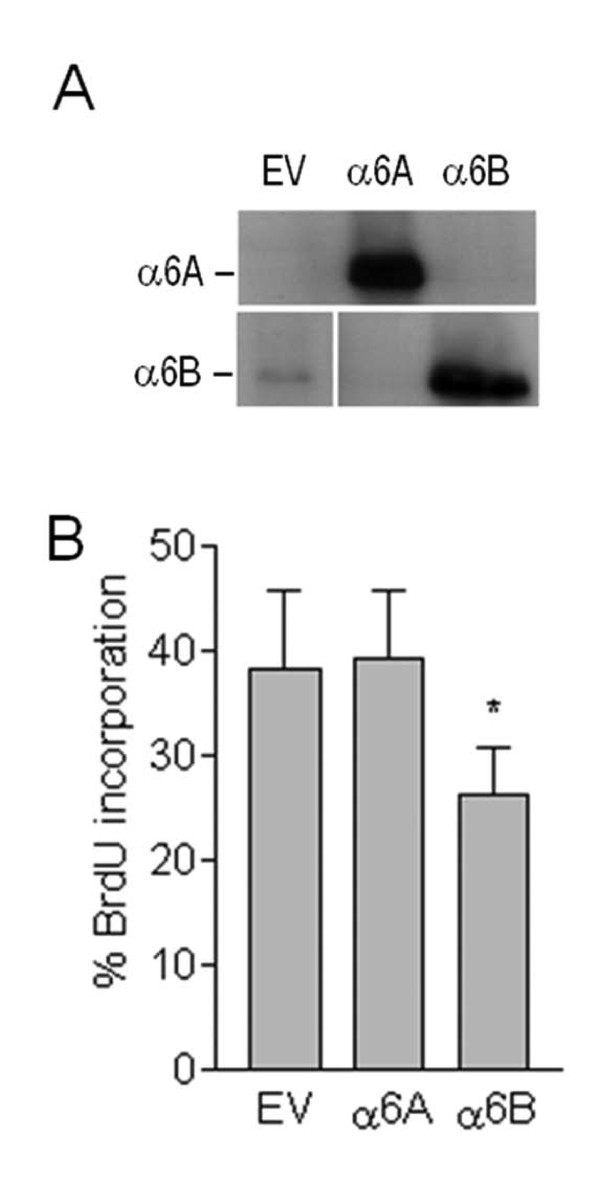
**Forced expression of the α6B subunit inhibits intestinal cell proliferation**. (A) Forced expression of the α6A and α6B integrin subunits was confirmed by western blot. (B) Quantification of Caco-2/15 cells undergoing S phase entry expressing α6A and α6B vs an empty vector control was assessed by BrdU incorporation. Mean ± SD. *: Statistically significantly different from the A variant, *p *< 0.01, Tukey's One Way Analysis of Variance (ANOVA).

**Figure 6:**
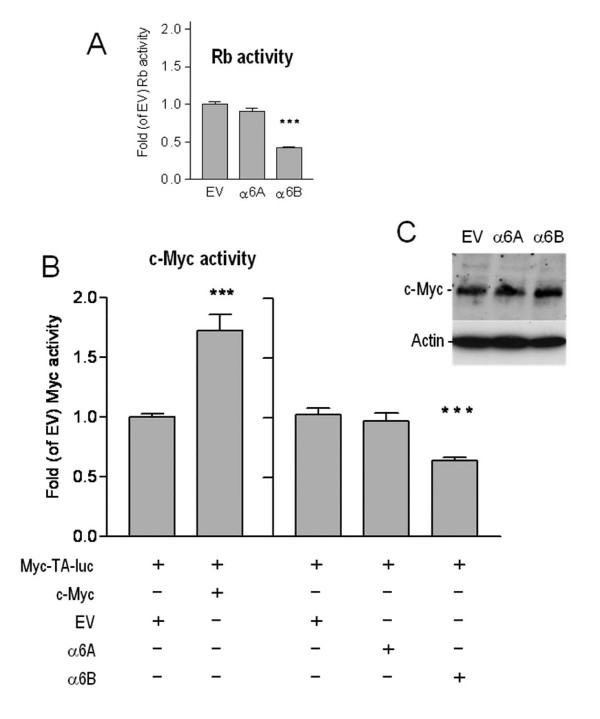
**Response of Rb and c-Myc promoter activities in Caco-2/15 cells**. (A) Rb activity was determined with an Rb-TA-Luc responsive promoter in the presence of forced α6A or α6B expression. (B) c-Myc activity was determined with a Myc-TA-luc responsive promoter. Left: Effect of forced c-Myc expression on the myc-responsive promoter. Right: Effect of forced α6A or α6B expression on the myc-responsive promoter. Mean ± SEM. ***: statistically significantly different from empty vector (EV), *p *< 0.001, Tukey's One Way Analysis of Variance (ANOVA). (C) Representative western blot for c-Myc expression in Caco-2/15 cells stably expressing the α6A and α6B integrin subunit or EV as in Figure 5.

We consequently opted to perform studies on nucleofected cell populations over-expressing the two variants despite the difficulty in maintaining the α6B over-expressing cells, analyzing the cells shortly following the 10-day antibiotic selection period ensuring that all cells expressed their respective episomal vectors (Figure 5A). Proliferation was assessed using BrdU incorporation assays. With this strategy we consistently observed that the proportion of α6B transfectants entering S-phase was significantly diminished compared to the α6A transfectants and the empty vector control (Figure 5B), suggesting that predominant expression of the α6B subunit inhibits S-phase entry in intestinal epithelial cells in accordance with the predominant expression of this subunit in the non-proliferative compartment of the colon and it's relative down-regulation in colon carcinomas. Consistent with these observations, a specific reduction in transcriptional Rb activity was observed in luciferase assays (Figure 6A) supporting the observation that the α6B-expressing cells are exiting cell cycle in G1 [29].

### α6B inhibits c-Myc activity in colon cancer cells

Colon cancer cells and glandular proliferative cells require the activity of the proto-oncogene c-Myc for proliferation [30]. We next evaluated the ability of the α6B subunit to modulate c-Myc activity by performing co-transfections of the two integrin α6 variants with a luciferase reporter plasmid responding to c-Myc activity (pMyc-Ta-Luc). Experiments performed in colon cancer cells (Caco-2/15) demonstrated that pMyc-TA-luc activity was significantly down-regulated by α6B over-expression (Figure 6B), but was not altered by α6A. The decrease in c-Myc activity in the α6B co-transfections was not due to any changes in c-Myc protein levels as determined by western blot analyses (Fig 6C).

## Discussion

The functional importance of the instructive interactions between the epithelial cell compartment with the surrounding extracellular milieu has long been recognized in the intestinal tract [31,32]. Our observations that the α6A and α6B splice variants are differentially expressed in progenitor and mature cells of the colonic epithelium, located in the lower and upper half of the glands respectively [33], are consistent with previous findings showing the existence of distinct microenvironments between the proliferative and differentiation compartments in the gut [7,34]. More importantly, it suggests that the intrinsic role of each α6 variant is distinct. In this context, our finding that the α6B variant exclusively repressed proliferation concurs with its low level of expression in the proliferative zone and its predominance in the non-proliferative compartments of the colon as shown herein, as well as in the small intestine [35]. The effect of the α6B variant on cell proliferation *in vitro *appeared sufficient to prevent expansion of these cultures although the potential low grade contribution of apoptosis or autophagy cannot be excluded at the present time.

Colon cancer is the third leading cause of cancer related mortality in the United States [36]. The occurrence and development of most colon cancers follows a stereotypical pattern of a progressive accumulation of gain- and loss of function mutations of oncogenes and tumor suppressor genes [37]. It is also clear that neoplastic cells tend to up-regulate the expression of integrins favoring their migration, survival and proliferation during the complex multistep generation of tumors [2]. The mechanistic and biochemical roles of the α6β4 integrin in carcinoma biology are well documented[4,38]. Our results describe novel aspects of the biology of the α6β4 integrin variants in colon cancer. Thus, after having identified that distinct forms of the integrin β4 subunit were expressed in normal intestinal proliferative vs cancer cells [8], we observed herein a shift in α6 variant expression from a predominantly high α6B/α6A ratio in the normal colon to a predominantly low α6B/α6A ratio in primary tumors.

c-Myc is a key player in cancer formation and progression due to the numerous roles it plays in proliferation control and apoptosis [39]. Indeed, c-Myc expression has been demonstrated to be up-regulated in 70% of colon cancers [40] and its well documented effects include stimulation of cyclinD expression, inhibition of p21^cip1 ^and p27^kip1 ^expression and inactivation of Rb, leading to enhanced G_1 _to S-phase transition [39]. Given the central role of c-Myc in the control of critical cellular events, its transcriptional functions are tightly regulated by several molecular pathways [41]. Prototypically, the control of cellular c-Myc levels are largely attributed to the canonical activity of the Wnt-pathway [42]. However, the activity and expression levels of c-Myc are regulated by several other molecular mechanisms including distinct signalling pathways and interaction with binding partners [39,43]. For instance, the nucleoshuttling scaffold protein bridging integrator-1 (Bin1) has been shown to strongly inhibit c-Myc transcriptional activity in a Wnt-pathway independent manner [44,45]. Interestingly, Bin1 can selectively interact with the cytoplasmic domain of the α6B integrin subunit in yeast two-hybrid studies [46] and its expression has been reported to be associated with the non-proliferative cell population in the normal intestine [47] suggesting a possible mechanism for the inhibitory effect of α6Bβ4 on c-Myc activity.

While there is substantial evidence for the differential capacity of the α6Aβ1 and α6Bβ1 integrins to initiate intracellular signalling [15,16] and facilitate migration on laminin [15], these studies have all found that the α6Aβ1 integrin functions as the "active" integrin, whereas the α6Bβ1 integrin appears to have no major active role in these events [15,16,48-50]. In this context it is noteworthy that over-expression of the α6A variant in colon cancer cells did not stimulate proliferation as compared to the control cells, but rather the α6B variant actively inhibited proliferation and c-Myc activity, under conditions where integrin/ligand interactions occurred. It is furthermore noteworthy that the α6A/B splice variants can dimerize with both the β1 and the β4 subunits to form two distinct functional integrins, α6β1 and α6β4, but that the α6 subunit preferentially dimerizes with the β4 subunit [7,13,26], as confirmed herein. Thus, the specific ability of the α6B subunit to inhibit proliferation, in accordance with its predominant expression in the quiescent compartment of the normal colon and its down-regulation in primary colon cancers and adenocarcinoma cell lines, strongly suggests that the expression and ratio of the α6A and α6B splice variants are inherent to normal intestinal homeostasis and exploited by colon cancer cells.

## Conclusion

In this study, we provide the first functional evidence that the α6Bβ4 integrin can exert distinct biological functions compared to its α6Aβ4 counterpart. Analyzing the expression of the two α6 variants in the human normal colon led to the discovery that the A and the B variants are differentially expressed in proliferative and differentiated cells, respectively. More significantly, a net shift toward a predominant A variant expression was observed in primary colorectal tumors and in adenocarcinoma cell lines suggesting that a high α6A/B ratio is required for both normal and cancer cell proliferation. To test this hypothesis, we forced α6A and α6B expression and found that re-establishing predominant α6B subunit expression inhibited colon cancer cell proliferation and c-Myc activity. Taken together, these findings point out the importance of integrin variant expression in colon cancer cell biology.

## Competing interests

The authors declare that they have no competing interests.

## Authors' contributions

ABD carried out most of the cell and molecular biology studies and drafted the manuscript. ICT carried out the immunofluorescence and related experimental set-up. JFG participated in the luciferase assays and immunofluorescence of the colon cancer specimens. NB carried out the immunoprecipitation studies and participated in the writing of the manuscript. FP participated with the luciferase and BrdU assays. RG performed the proliferation studies. DJ designed the episomal vectors. JFB conceived the study, participated in its design and coordination and helped to draft the manuscript. All authors read and approved the final manuscript.

## Pre-publication history

The pre-publication history for this paper can be accessed here:

http://www.biomedcentral.com/1471-2407/9/223/prepub
